# Development, Optimization, Characterization, and Application of Electrochemical Biosensors for Detecting Nickel Ions in Food

**DOI:** 10.3390/bios11120519

**Published:** 2021-12-16

**Authors:** Liliana Anchidin-Norocel, Wesley K. Savage, Gheorghe Gutt, Sonia Amariei

**Affiliations:** 1 Faculty of Medicine and Biological Sciences, Stefan cel Mare University of Suceava, 720229 Suceava, Romania; liliana.norocel@usm.ro; 2 Integrated Center for Research, Development and Innovation in Advanced Materials, Nanotechnologies, and Distributed Systems for Fabrication and Control, Stefan cel Mare University of Suceava, 720229 Suceava, Romania; 3 Faculty of Food Engineering, Stefan cel Mare University of Suceava, 720229 Suceava, Romania; g.gutt@fia.usv.ro (G.G.); sonia@usv.ro (S.A.)

**Keywords:** antibodies, aptamers, synthetic receptors, biosensors, immobilization process, screen-printed electrodes

## Abstract

Nickel is naturally present in drinking water and many dietary items, which expose the general population to nickel ingestion. This heavy metal can have a variety of harmful health effects, causing allergies and skin disorders (i.e., dermatitis), lung, cardiovascular, and kidney diseases, and even certain cancers; therefore, nickel detection is important for public health. Recent innovations in the development of biosensors have demonstrated they offer a powerful new approach over conventional analytical techniques for the identification and quantification of user-defined compounds, including heavy metals such as nickel. We optimized five candidate nickel-biosensing receptors, and tested each for efficiency of binding to immobilization elements on screen-printed electrodes (SPEs). We characterized the application of nickel-detecting biosensors with four different cultivated vegetables. We analyzed the efficiency of each nickel-detecting biosensor by potentiostat and atomic absorption spectrometry and compared the results from the sample analytes. We then analyzed the performance characteristics and responses of assembled biosensors, and show they are very effective at measuring nickel ions in food, especially with the urease-alginate biosensor affixed to silver SPEs, measured by cyclic voltammetry (sensitivity—2.1921 µA Mm^−1^ cm^−2^ and LOD—0.005 mg/L). Given the many advantages of biosensors, we describe an optimization pipeline approach to the application of different nickel-binding biosensors for public health, nutrition, and consumer safety, which are very promising.

## 1. Introduction

We are becoming increasingly aware of elements in the environment that affect human growth, development, and health, through air, water, and food. As the global population grows, increasing demands on drinking water and food supply require more efficient, higher-throughput, and more cost-effective solutions to monitor food production systems that ensure consumer safety. In addition to synthetic compounds, many naturally-occurring elements can accumulate in production systems that, even at low concentrations, can have chronic health effects [1,2,3,4]. Nickel (Ni) is an example case because it is a prevalent element in the environment that is naturally found in water and food. It can enter the body by inhalation, ingestion, and dermal absorption, and is known to cause a variety of adverse health effects, including contact dermatitis, lung fibrosis, cardiovascular and kidney diseases [5,6,7]. There is also evidence that some nickel compounds are carcinogens, and have been linked to lung and nasal cancers [8]. Due to its widespread presence in drinking water and food products, an alarming number of people face long-term exposure to Ni that can lead to health problems even at relatively low levels. This is a public health matter that can easily be monitored with affordable and efficient testing.

The prevalence of nickel sensitivity varies from 4 to 13.1% in different countries, and an estimated 12.8% of people worldwide have a nickel allergy [9,10]. Given the natural prevalence of nickel and its effects on human health, measuring nickel consumption is rather important. However, the precise measurement of daily nickel intake from food and drink presents numerous technical challenges because the chemical form of nickel determines how it enters the body. Health concerns over trace elements in foods such as nickel have led to a growing interest in the development and deployment of biosensors for applications in food analysis [11,12,13,14,15,16,17]. This novel enterprise involves coordination of knowledge and practice in food chemistry, analytical chemistry, biochemistry, biotechnology, and materials science, among other disciplines. For many reasons, biosensors are valuable tools in the food industry because of the many applications for protecting consumer safety [18]. 

Traditional reference methods that measure total nickel content include flame atomic absorption spectrometry (FAAS), graphite furnace atomic absorption spectrometry (GFAAS), inductively coupled plasma optical emission spectrometry (ICP-OES), and mass spectrometry (ICP-MS) [19,20,21,22]. These methods can detect Ni levels in food and water samples, and require preconcentration or separation steps. Other methods used for nickel quantification include spectrophotometric techniques (ultra-violet [UV]-visible absorption, photodiode array), total reflection X–ray fluorimetry (TXRF), flow injection atomic absorption, and electrochemical methods [5,23]. These methods, while functional, are restrictive because they are time-consuming, costly, and require complex sample preparations and technical expertise to operate [24].

Modern improvements in heavy metal detection employ electroanalytical methods that include ion-selective electrodes, anodic stripping voltammetry (ASV), polarography, and other voltammetric methods. These detection methods do not require preconcentration or separation steps to analyze samples, and thereby reduce the preprocessing steps required by traditional reference methods. Electrochemical sensors are instruments in which an electrode is used as a transducer element. These have wide applicability for hazardous, ecological, and environmental assessments in various industrial applications, such as manufacturing, horticulture, medicine, biotechnology, quantitative analytics, and natural products industries. Electrochemical biosensors present a significant number of advantages, because they can identify specific targets with high selectivity and affinity. They are also inexpensive to produce, have low toxicity, are easily chemically modified/labeled, can be screened in vitro, yield accurate and reproducible data, and have a lack of immunogenicity [25]. Two types of electroanalytical sensors commonly used to measure analytes are potentiometric and amperometric sensors. These methods essentially convert the binding of an analyte of interest into an electrical signal that is captured and quantified from a sample. They can accomplish a wide range of analytical tasks that require rapid, sensitive, and inexpensive detection and quantification by way of biochemical reactions that involve the binding of molecules or elements [26]. The drawbacks to this technique are that they can suffer from insensitivity to metal ion speciation; therefore, they may not be suitable for many situations; they also require sample destruction, which may not be useful in some applications. However, there are opportunities to improve these techniques and develop methods that are more sensitive, accurate, and require minimal sample destruction [27]. Biosensors offer promise in this regard because they overcome the drawbacks of existing methods, and given that they can detect metal ions without excessive sample destruction, they hold potential for broader applications in the consumer food industry. Another benefit of biosensors is that, although screen-printed electrodes immobilized with receptors are single-use, they can be reformatted, reprinted, and reused.

While biosensors have many advantages over legacy techniques, the challenge in developing biosensors is in finding the appropriate biologically-active element that selects for a single component (a target analyte) from a matrix of compounds found in natural and processed food products. Thus, the limitation in this application is that the analyte must be a known compound, with an architecture that a biosensor can be specially constructed to detect. Biosensors also require selecting efficient methods for immobilizing bioreceptors on surfaces of screen-printed electrodes (SPEs) to ensure that the biological elements are affixed and intact; otherwise, if compromised, they will fail to detect target compounds in sample analytes [28].

Currently, the broad array of selective recognition options for detecting metal ions by synthetic receptors has attracted great interest for various applications. Synthetic receptors (molecules with functional groups able to selectively interact with an analyte) are more stable than biological ones, have a relatively low molecular weight, and bind more efficiently to analytes [29]. Aptamer-based biosensors can recognize specific targets for in vitro applications to detect heavy metals, and have been applied to detect biological compounds such as malaria proteins [30,31].

Dimethylglyoxime (DMG) is the most widely-used synthetic ligand for nickel biosensors [32,33]. It is used with Adsorptive Cathodic Stripping Voltammetry [34,35,36] and Adsorptive Cathodic Square-Wave Stripping Voltammetry, producing dynamic ranges for measurement of 3–12 µg/kg [37], 5–175 µg/kg [38], and 5.9–35.2 µg/kg [39]. Square Wave Adsorptive Stripping Voltammetry has also been used for nickel detection with nioxime (producing a dynamic range of 0.11–0.58 µg/kg [40]), and with nioxime and hemisodium salt of N-[2-hydroxyethyl] and piperazine-N′-[2-ethanesulfonic acid] [41]. DNAzyme-CdSe nanocomposite has been used as a bioreceptor by differential pulse anodic stripping voltammetry, obtaining a dynamic range of 1.173 µg/kg–11.73 mg/kg and a low limit of detection (6.67 nM) [42]. With the same method, calconcarboxylic acid has demonstrated a far greater range of detection (0.1–28 µg/kg) [43]. Other techniques use potentiometry with biosensors such as *Bacillus sphaericus* MTCC 5100, yielding urease enzyme [5], (E)-N′-Chlorobenzylidene-4-methylbenzenesulfonohydrazide [44], 2-hydroxy-1-naphthylidene-N-cyanoacetohydrazone, and sodium diethyldithiocarbamate [45]. 

Aptamers are biological ligands that can fold into distinct secondary and tertiary structures, and bind targets with high affinity (i.e., dissociation constants on the order of nano- to picomolar). They can recognize their targets with high specificity, similar to how binding between antibodies and antigens forms tight complexes [46]. Antibodies can be utilized in biosensors because of the specific binding properties they exhibit. There are five classes of antibodies based on structure and function, and among them, IgG is most frequently used for heavy metal detection because of its high binding affinity and specificity to metal species. Other biosensors utilize monoclonal, polyclonal, and recombinant antibodies as ligands in receptors, dependent on the specific application. [47]. Biological ligands, including antibodies and aptamers, are part of the future of biosensor developments because of their specificity for binding biologically-relevant compounds.

Here, we describe a biosensor technique to detect nickel ions by two voltammetric methods, and discuss receptor optimization and immobilization of elements for analyte discrimination using SPEs with biological and synthetic ligands. Our goal is to describe biosensors for nickel detection with analytical results that characterize the applicability of a heavy metal biosensor on different vegetable samples. The novelty of this work is the potential adoption of biosensors for detecting heavy metals in food products, which have immense applicative value in food production and public health. Most nickel biosensors described to date have only been used to analyze the performance of sensors with aqueous solutions, without practical applications to food analysis. The performance of biosensors in food is important to quantify, because analyte concentrations vary in food items and require useful detection and sensitivity limits.

## 2. Materials and Methods

### 2.1. Chemicals

We used the following commercial compounds and analytical reagents to construct and characterize five biosensor variants for nickel detection on samples from four types of vegetables: nickel sulphate (0.1–10 mg/L); dimethylglyoxime (DMG; 0.1 mol L^−1^); protein A-agarose; benzophenone (1%); alginate (1%); urease (0.1%); ethylenediamine (1%); EtOH (70%); ammonium buffer solution; and nitric acid (1%) (Sigma Aldrich Chemical Corp., St. Louis, MO, USA). We obtained screen-printed electrodes (SPEs) based on carbon (DRP110), bismuth (DRP-110Bi) and silver (DRP-110-AAGNP) inks (dimensions: 3.4 × 1.0 × 0.05 cm^3^) from Metrohm DropSens (Metrohm Corp., Herisau, Switzerland). All types of SPEs we used has a single 4 mm diameter working electrode and a counter electrode made of carbon, optimized to work with 50 μL volumes of analytes. We mixed all solutions with deionized water (resistivity of 18.2 M; Millipore Direct-Q 3 UV, Millipore Corp., Burlington, MA, USA). 

### 2.2. Electrochemical Measurements

We performed two types of electrochemical measurements with a Metrohm Autolab bipotentiostat µStat 300 using DropView 8400 software v. 3.6 20B0514 (Metrohm Corp. Herisau, Switzerland). We conducted SPE tests with both Linear Sweep Voltammetry and Cyclic Voltammetry with the below-specified conditions. We measured nickel concentration with electrolytes supported by an ammonium buffer solution, and delivered 50 μL of solution on the surface of working electrodes with different receptors to form nickel complexes. All voltammetric measurements were carried out at room temperature (20–25 °C).

#### 2.2.1. Linear Sweep Voltammetry (LSV)

We set the LSV parameters in the following steps: an initial potential of 0 V, an end potential 0.9 V, a step potential of 0.002 V, and a scanning rate of 0.05 V/s. 

#### 2.2.2. Cyclic Voltammetry (CV)

We conducted cyclic voltammetry with the following conditions: initial potential of 0.1 V, switching potential 0.9 V, a step potential of 0.002 V, and a scanning rate 0.05 V/s. 

### 2.3. Receptor Immobilization on Screen Printed Electrodes (SPEs)

We tested five immobilization receptor combinations for functional biosensing interactions with nickel in food analytes: (1) dimethylglyoxime and alginate, (2) dimethylglyoxime and benzophenone, (3) urease and alginate, (4) protein A-agarose, and (5) ethylenediamine and alginate. We immobilized dimethylglyoxime on SPEs with both benzophenone (UV condition at 365 nm) and an alginate solution, and also used an alginate solution to immobilize urease, protein A-agarose, and ethylenediamine on SPEs.

### 2.4. Testing Biosensor Performance on Nickel Detection in Food

We tested the sensitivity of nickel-detection biosensors on four commercially available cultivated vegetables using the above two voltammetric methods: (1) mushrooms (*Armillaria mellea*), (2) zucchini (*Cucurbita pepo*), (3) red radish (*Raphanus sativus*), and (4) white potato (*Solanum tuberosum*). We prepared the analytes by homogenizing 1.0 g of sample in 10 mL of buffer solution, and delivered 50 μL of homogenate derived from the samples onto the surface of working receptor-immobilized SPEs. We conducted tests for each sample three times, using SPEs configured with new electrodes for each test.

### 2.5. Atomic Adsorption Spectrometry Method (AAS)

We determined nickel content in food samples by the Atomic Adsorption Spectrometry (AAS) method described by Ferreira, et al., 2001 [48]. We used a starting sample mass of 1.0 g that was decomposed at 500 °C for 60 min, followed by treatment with either concentrated nitric acid or 30% (*v*/*v*) hydrogen peroxide, and by a second heating step of 45 min. We used the AAS method for comparing the biosensor results to back test the similarity of the data.

## 3. Results

### 3.1. Protein A-Agarose and Nickel(II) Voltammetric Measurements

Nickel-binding proteins play an important role in many biological compounds and processes, meaning that these nickel-associated properties of biologics can be utilized in other biosensing applications (e.g., biotechnology, food science, agriculture, and food production). In addition to enzymes and proteins, Ni(II) is found in nucleic acids and their components, and can be used to probe nucleic acid structure [49]. These different capacities of Ni confirm that that it is able to form stable complexes with many kinds of coordination ligands, a property that is reflected in the great diversity of Ni(II) receptors found in biological systems [31,50]. Moreover, because of its native binding affinities for biological compounds, Nickel (Ni) can be bound to agarose beads by chelation, allowing for the examination of interactions between nickel and Protein A-agarose. Agarose is a polysaccharide obtained from seaweed, with a particular structure that allows spontaneous gelation. Agarose-based beads are highly porous, mechanically resistant, chemically and physically inert, and hydrophilic. These features—that could be further improved by means of covalent crosslinking—render it particularly suitable for enzyme and protein immobilization [51]. For this reason, we used Protein A-agarose as a receptor affixed on SPEs to bind Ni from sample analytes with both LSV and CV (Figure S1 in Supplementary Materials). Figure S1a illustrates the cyclic voltammograms curves obtained with Protein A-agarose (for all standard nickel solutions) immobilized on carbon SPEs, and shows the absence of redox peaks, suggesting a high charging/discharging rate capability of the electrode and pure electric double-layer capacitance.

Biosensors constructed with bismuth and silver SPEs yielded slightly visible peaks for Ni detection analyzed by cyclic voltammetry (Figure S1b,c). Linear sweep voltammetry detected a low background current in the potential range from 0.10 to 0.90 V for carbon and bismuth SPE biosensors, compared with silver electrodes that showed a current above 0.10 V, indicating that Ni began binding the biosensor SPE (Figure S1d–f). 

### 3.2. Dimethylglyoxime (DMG) and Nickel(II) Voltammetric Measurements

**Figure 1:**
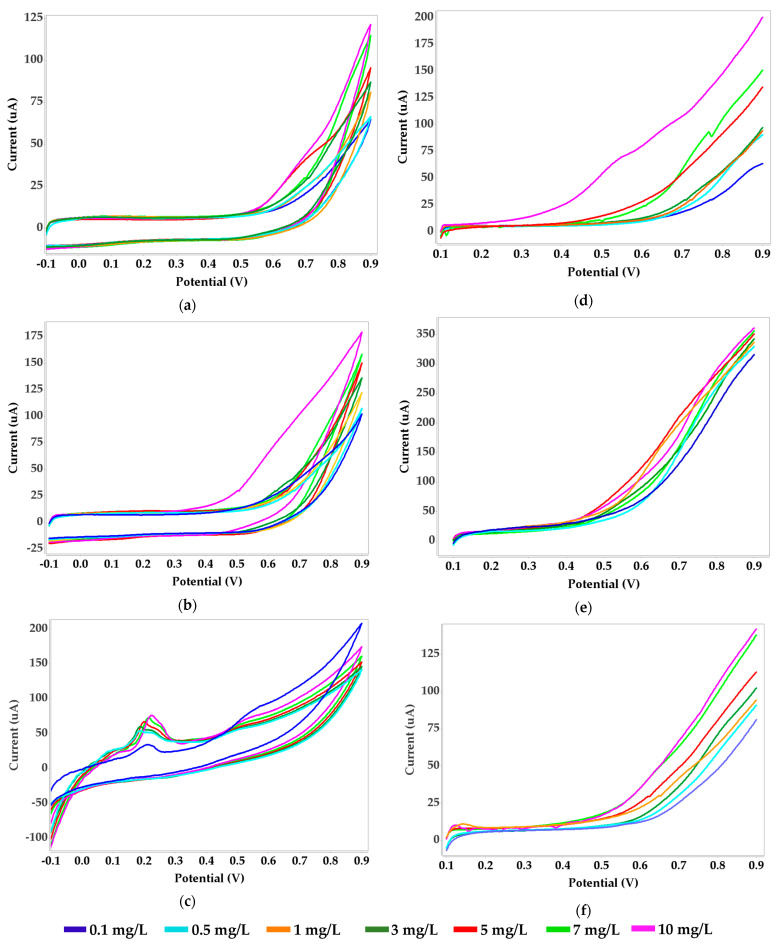
Voltammograms obtained for nickel standard solution and dimethylglyoxime immobilized with alginate; CV with SPEs: (**a**) carbon, (**b**) bismuth, (**c**) silver; LSV with SPEs: (**d**) carbon, (**e**) bismuth, (**f**) silver.

Dimethyglyoxime (DMG) is one of the most commonly used synthetic ligands that binds nickel (Ni) ions and forms a stable Ni-DMG complex. This complex is produced by the chelation of Ni ions with the organic ligand DMG in an alkaline–ammonia medium. The reaction involves two DMG molecules acting as chelating agents to form a Ni–DMG square–planar complex. This reaction is very sensitive, and with its stability it is useful as a confirmation test for the presence of Ni(II) cations, even in very low concentrations. DMG can adsorb to an electrode surface and significantly enhance the electrochemical response of Ni ions [52]. Because of its sensitivity and stability in forming Ni–DMG complexes, we used DMG affixed to SPE biosensors (i.e., carbon, bismuth and silver) immobilized with alginate and benzophenone in our experimental assays of Ni ions in four food types (Figure 1 and Figure S2).

#### 3.2.1. Dimethylglyoxime (DMG) Immobilized with Alginate

With this method, Ni(II) is complexed by DMG in solution and the resulting Ni(DMG)_2_ complex adsorbs and accumulates onto the surface of the SPEs, where Ni(II) can be further reduced by cathodic scan. The CV and LSV results are presented in Figure 1, illustrating the electrochemical behavior of a Ni–DMG complex that involves the continuous increase in the amount of current exchanged by a nickel standard solution with repeated cycling for carbon and bismuth electrodes. Screen-printed electrodes modified with silver show a redox peak that is proportional with nickel concentration. Linear sweep voltammetry shows very low background currents measured in the potential range from 0.10 to 0.90 V for all types of SPEs tested.

#### 3.2.2. Dimethylglyoxime (DMG) Immobilized with Benzophenone

The results of a second dimethylglyoxime immobilization were examined with benzophenone in UV light conditions, and the results of the electrochemical measurements with three types of SPEs are presented in Figure S2. Examination of these graphs indicates the lack of oxidation redox peaks, with the exception of the slight presence in the silver electrode (in cyclic voltammetry, Figure S2c). 

### 3.3. Urease and Nickel(II) Voltammetric Measurements

**Figure 2:**
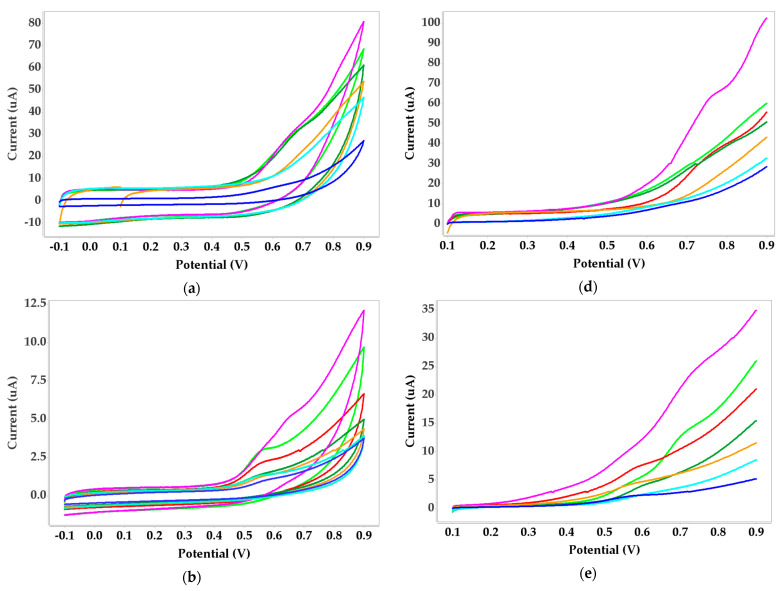

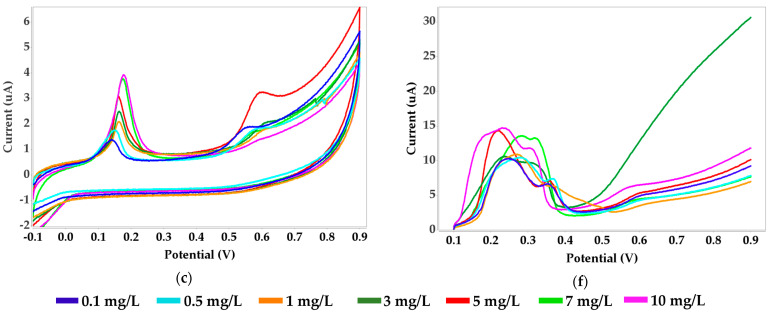
Voltammograms obtained for nickel standard solution and urease immobilized with alginate; CV with SPEs: (**a**) carbon, (**b**) bismuth, (**c**) silver; LSV with SPEs: (**d**) carbon, (**e**) bismuth, (**f**) silver.

Coordination chemistry of metal ions from known biological samples can be used to inform the design of selective heavy metal receptors. In the case of Ni(II), the coordination environments vary dramatically, perhaps reflecting the various roles of metal cofactors in nickel-dependent enzyme complexes [53]. Urease is a nickel-dependent enzyme found in many organisms, including fungi, algae, plants, and prokaryotes. It is very substrate-specific, and catalyzes the hydrolysis of urea into carbon dioxide and ammonia [54]. Because urease is a biologically relevant compound in natural organisms [55], we used it as the ligand to bind nickel with voltametric measurements. We present the results of the voltammetric behavior that displayed redox peaks for silver SPEs with CV and LSV in Figure 2.

### 3.4. Ethylenediamine and Nickel(II) Voltammetric Measurements

Because Ni(II) is relatively low in the metal spectrochemical/electrochemical series, chelating groups based on strong-field ligands can be incorporated into any of the four coordination sites of Ni(II). The chelating groups include pyridine, bipyridine, phenanthroline, ethylenediamine, ammonia, and carbonyls. Ethylenediamine is one of the more studied nitrogen-binding, synthetic ligands, forming a large number of complexes with many different metal ions (e.g., Ni, Fe, Cu Zn, and Pb). It usually acts as a bidentate ligand, but examples are known in which ethylenediamine acts either as a monodentate or a bridging ligand [26,32]. We used ethylenediamine for Ni(II) complexation by voltametric measurements and immobilization with alginate on SPE surfaces (Figure S3), and found that the interaction between nickel and ethylenediamine performed better with bismuth and silver SPE biosensors analyzed by cyclic voltammetry. The interaction between nickel and ethylenediamine was better explained with bismuth and silver SPE biosensors analyzed by cyclic voltammetry that yielded slightly visible peaks.

### 3.5. Analytical Performance Characteristics of the Five Biosensors

Table 1 lists the performance characteristics (i.e., sensitivity of nickel detection) of the five types of biosensors coupled to the three SPEs (i.e., carbon, bismuth, and silver). The results were obtained by cyclic and linear sweep voltammetry. Sensitivity was calculated using Equation (1) below:

Sensitivity = m/A(1)

where m is the slope of the calibration curve (µA Mm^−1^), and A is the area of active surface (cm^2^) [56].

The LOD was determined according to Norocel (2019), using the measured limit of the blank sample (LoB) and the standard deviation of the lowest sample concentration (Equation (2)) [57]:

LOD = LoB + SDlow concentration sample(2)

**Figure 3:**
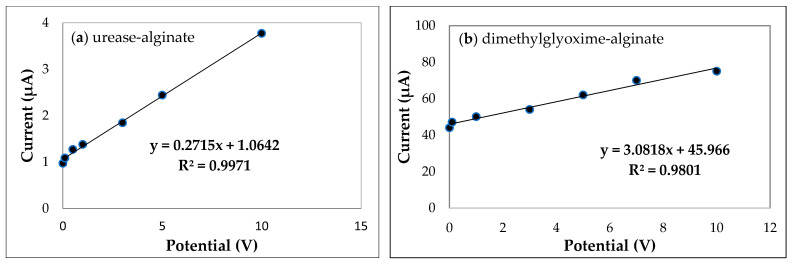
Calibration curve for cyclic voltammograms (**a**) biosensor with urease-alginate and (**b**) dimethylglyoxime-alginate.

**Figure 4:**
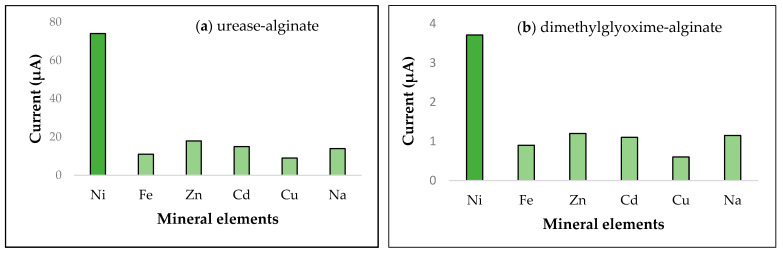
Selectivity evaluation (Current, µA) of two biosensor types against the interference of mineral elements: (**a**) urease-alginate biosensor; (**b**) dimethylglyoxime-alginate biosensor.

Calibration curves are the standards for evaluating the quantitative detection of a target of a biosensor. Figure 3 illustrates the calibration curves for the most efficient biosensors determined by redox peaks. The two most efficient were urease-alginate and dimethylglyoxime-alginate. All other calibration curves are displayed in Figures S4–S8. The specificity of a biosensor is also essential to validate its applicability for specific detection of a target, and we present specificities for six elements (i.e., nickel, iron, zinc, cadmium, copper, and sodium ions) in Figure 4. The results of the selectivity evaluation of the most efficient biosensors against the interference of minerals demonstrates that these developed methods exhibit good selectivity.

### 3.6. Biosensors Optimization

**Figure 5:**
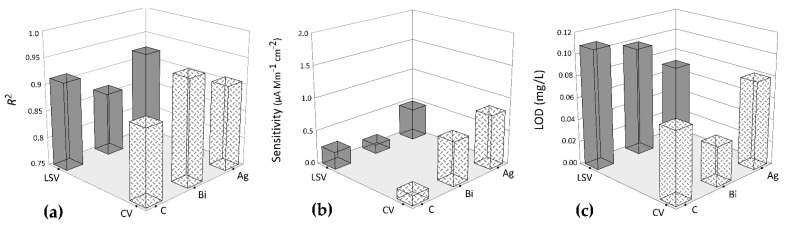
3D surfaces for performance parameters of three electrode types (C-carbon, Bi-bismuth, and Ag-silver) and two voltammetric techniques (CV and LSV): (**a**) R^2^, (**b**) Sensitivity (µA Mm^−1^ cm^−2^), and (**c**) Limit of Detection (LOD; mg/L).

We optimized five receptors with three types of SPEs by two voltammetric techniques (LSV and CV). We report the R^2^ values of the results, as well as the sensitivity and limit of detection (LOD) values, in Figure 5. ANOVA results for the quadratic model with linear regression (R^2^) were not significant (Figure 5a), implying that the overall mean may be a better predictor of the response than the null model. The quadratic model was significant for biosensor sensitivity (*p* = 0.0103) and LOD (*p* = 0.0102). Three-dimensional surface plots (Figure 5b) of biosensor sensitivity showed higher values for bismuth and silver electrodes analyzed by cyclic voltammetry. Silver was more sensitive at detection by linear sweep voltammetry. The LOD was greater for silver electrodes with LSV and for bismuth electrodes with CV (Figure 5c). The Desirability score (D) for optimization was 0.844 (ranges from suboptimal, 0, to 1.0, optimal) indicating that silver electrodes combined with urease ligands and the CV assay were the most efficient biosensor combinations for nickel detection.

### 3.7. Testing the Biosensors for Food Samples

The results for nickel content measurements from samples of mushrooms (*Armillaria mellea*), zucchini (*Cucurbita pepo*), red radish (*Raphanus sativus*), and white potato (*Solanum tuberosum*) were similar for the five biosensor types and both voltametric techniques, although the absolute measures differed in small degrees. In general, the biosensors performed better than the AAS method. The results are presented in Table 2, Table 3, Table 4 and Table 5.

## 4. Discussion

Over all methods tested, both cyclic voltammetry and linear sweep voltammetry showed redox peaks for the silver screen-printed electrode (SPE) that indicate it is the most sensitive SPE for nickel detection in the cultivated vegetable samples we assayed. A well-defined Ni(II) peak around 0.2 V increased proportionally with nickel concentration using the silver SPE affixed with urease-alginate (Figure 4a). Redox-peaks were also evident by both voltammetric methods for nickel detection on SPEs affixed with dimethylglyoxime-alginate, although the results were less clear (Figure 2c). For other types of SPEs, voltammograms either did not produce redox peaks, or the peaks were very low, noting that the potentials differed for the determination of LODs depending on the biosensor analyzed. Biosensor sensitivities, summarized in Table 1, indicate that the urease-alginate combination produced the highest sensitivity (2.1921 µA Mm^−1^ cm^−2^) with cyclic voltammetry and the silver SPE. Protein A-agarose was less sensitive to nickel (1.4753 µA Mm^−1^ cm^−2^) with the same electrode and analytical technique. The highest LOD scores were obtained for the same biosensors: 0.005 mg/L for urease-alginate, and 0.019 mg/L for Protein A-agarose. Linear sweep voltammetry produced lower sensitivity values compared with cyclic voltammetry (0.6584 µA Mm^−1^ cm^−2^ for Protein A-agarose and 0.6978 µA Mm^−1^ cm^−2^ for dimethylglyoxime–benzophenone). LODs from Table 1 for these biosensors were also lower (0.012 mg/L for Protein A-agarose, and 0.060 mg/L for dimethylglyoxime-benzophenone).

Performance characteristics suggested that the urease receptor affixed to a silver SPE was the most sensitive at detecting differences in total nickel content for the four vegetable samples we assayed. Although the dimethylglyoxime-alginate and Protein A-agarose affixed to carbon and silver SPEs were also effective for nickel detection, these biosensor combinations produced lower LOD scores. Nickel content in the mushroom samples (*Armillaria mellea*) analyzed by AAS was 7.98 mg/kg (Table 2). The best results obtained were by LSV for the biosensor constructed with the bismuth SPE and dimethylglyoxime-alginate (8.03 mg/kg), followed by the urease-alginate biosensor on the silver SPE (8.11 mg/kg). Similar results were obtained by cyclic voltammetry for the urease-alginate and bismuth SPE (8.16 mg/kg). Nickel content in zucchini (*Cucurbita pepo*) assayed with the AAS method was 0.80 mg/Kg. We found similar values for two biosensors analyzed by cyclic voltammetry (the silver SPE with urease-alginate, 0.84 mg/kg) and linear sweep voltammetry (the carbon SPE with urease-alginate, 0.78 mg/kg), suggesting mixed results. Assays for nickel content in red radish (*Raphanus sativus*) samples conducted with the AAS method produced a value of 7.11 mg/kg. In comparison, the biosensors were more effective at detecting nickel levels. The urease-alginate—silver SPE detected nickel levels of 7.18 mg/kg by cyclic voltammetry, and for both biosensor combinations with dimethylglyoxime-alginate—bismuth SPE and urease-alginate—silver SPE, the nickel content was measured at 7.28 mg/kg by linear sweep voltammetry. White potato (*Solanum tuberosum*) assays with the AAS method were 2.35 mg/kg, while urease-alginate biosensors yielded 2.41 mg/kg by cyclic voltammetry on the silver SPE, and 2.49 mg/kg by linear sweep voltammetry on the carbon SPE, indicating greater sensitivities of biosensors.

There are many studies that discuss the applications of electrochemical and optical biosensors to detect nickel ions, but only a few are specific to food applications. While there are a number of different methods and techniques in use for the determination of nickel ion content, the differences among them are determined by the required analytical performance characteristics, i.e., the detection limit, sensitivity, and dynamic range. Verma & Singh (2006) developed a sensor for nickel ions in food but did not specify the sensitivity; they reported only the range of Ni(II) detection: 0.002–0.04 ppb with a response time of 1.5 min, and reliability of 91.5% and 90.6% [5], values which cannot be compared with our biosensors. Alves et al. (2013) determined nickel content by adsorptive cathodic stripping voltammetry and obtained a LOD of 0.6 µg/L, but the sensitivity was not presented [58]. Padilla et al. (2021) recently described several types of biosensors used for nickel detection at trace levels with large-scale applications [59]; however, trace levels were not in the linear range found in food products, which points to the value that the biosensors we described here hold for applications in the food industry, and for public health.

## 5. Conclusions

Biosensors are ideal tools for detecting analytes including nickel because they can be synthesized to have very high specificity, and can be adapted for high-throughput processing. They are generally lower cost and portable, which makes them valuable for small-scale and mobile/remote applications. Most existing biosensors developed for nickel ion detection have limited application to industrial food production because they are designed to detect elements in trace levels (e.g., ppb). If existing biosensors and newer methods are deployed to measure heavy metal ions in food, it would greatly benefit consumer health because of the known toxic and/or adverse health effects of metal ion consumption. Our results suggest that biosensors can be readily developed for determining nickel ion concentration in food. We demonstrated this by the performance characteristics we measured in comparison with legacy reference methods (AAS). In particular, biosensors affixed with silver and carbon electrodes are effective for nickel detection (indicated by cyclic voltammetry). We found that urease-alginate, Protein A-agarose, and dimethylglyoxime-alginate were the most effective ligands for immobilizing analytes, which suggests that these biosensor combinations hold promise for wide-scale applications for the quantification of nickel ions in food.

## Figures and Tables

**Table 1:** Analytical performance characteristics of the five biosensors analyzed. LSV = Linear Sweep Voltammetry, CV = cyclical voltammetry.

Nickel Biosensor	SPE	Technique	R2	Sensitivity [µA Mm^−1^ cm^−2^]	Limit of Detection (LOD) [mg/L]
Protein A-agarose	Carbon	CV	0.9376	1.4753	0.050
	Bismuth		0.9242	0.4038	0.018
	Silver		0.9742	1.8433	0.019
	Carbon	LSV	0.9261	0.5189	0.090
	Bismuth		0.8607	0.4064	0.026
	Silver		0.9329	0.6584	0.012
Dimethylglyoxime -alginate	Carbon	CV	0.9779	0.1444	0.010
	Bismuth		0.9605	0.0984	0.028
	Silver		0.9801	0.2850	0.080
	Carbon	LSV	0.9556	0.0575	0.092
	Bismuth		0.8516	0.1720	0.098
	Silver		0.9482	0.1165	0.050
Dimethylglyoxime- benzophenone	Carbon	CV	0.7824	0.1107	0.100
	Bismuth		0.8751	0.0682	0.150
	Silver		0.8678	0.5175	0.092
	Carbon	LSV	0.9415	0.0769	0.120
	Bismuth		0.9574	0.0815	0.100
	Silver		0.9757	0.6978	0.060
Urease-alginate	Carbon	CV	0.8569	0.1725	0.050
	Bismuth		0.9782	0.8737	0.020
	Silver		0.9971	2.1921	0.005
	Carbon	LSV	0.9112	0.1099	0.040
	Bismuth		0.9846	0.2767	0.026
	Silver		0.9226	0.2562	0.030
Ethylenediamine -alginate	Carbon	CV	0.9343	0.1066	0.050
	Bismuth		0.9578	0.0812	0.100
	Silver		0.9328	0.1513	0.080
	Carbon	LSV	0.8831	0.1388	0.076
	Bismuth		0.9430	0.2005	0.050
	Silver		0.9112	0.1382	0.080

**Table 2:** Nickel content in mushrooms (*Armillaria mellea*).

Biosensor for Ni	SPEs	Technique	Nickel Content [mg/kg]	AAS [mg/kg]
Protein A-agarose	Carbon	CV	8.68	7.98
	Bismuth		8.23	
	Silver		8.32	
	Carbon	LSV	8.52	
	Bismuth		8.60	
	Silver		8.18	
Dimethylglyoxime-alginate	Carbon	CV	8.38	
	Bismuth		8.44	
	Silver		8.56	
	Carbon	LSV	8.26	
	Bismuth		8.03	
	Silver		8.17	
Dimethylglyoxime-benzophenone	Carbon	CV	8.76	
	Bismuth		8.25	
	Silver		8.81	
	Carbon	LSV	8.48	
	Bismuth		8.74	
	Silver		8.16	
Urease-alginate	Carbon	CV	8.29	
	Bismuth		8.16	
	Silver		8.51	
	Carbon	LSV	8.35	
	Bismuth		8.42	
	Silver		8.11	
Ethylenediamine-alginate	Carbon	CV	8.31	
	Bismuth		8.62	
	Silver		8.34	
	Carbon	LSV	8.50	
	Bismuth		8.64	
	Silver		8.43	

**Table 3:** Nickel content in zucchini (*Cucurbita pepo*).

Biosensor for Ni	SPEs	Technique	Nickel Content [mg/kg]	AAS [mg/kg]
Protein A-agarose	Carbon	CV	0.92	0.80
	Bismuth		1.26	
	Silver		1.02	
	Carbon	LSV	0.89	
	Bismuth		0.98	
	Silver		1.14	
Dimethylglyoxime-alginate	Carbon	CV	0.91	
	Bismuth		0.72	
	Silver		0.88	
	Carbon	LSV	0.98	
	Bismuth		1.22	
	Silver		1.28	
Dimethylglyoxime-benzophenone	Carbon	CV	1.26	
	Bismuth		1.08	
	Silver		1.10	
	Carbon	LSV	1.32	
	Bismuth		1.18	
	Silver		1.04	
Urease-alginate	Carbon	CV	0.97	
	Bismuth		0.96	
	Silver		0.84	
	Carbon	LSV	0.78	
	Bismuth		0.90	
	Silver		0.86	
Ethylenediamine-alginate	Carbon	CV	0.92	
	Bismuth		0.98	
	Silver		1.28	
	Carbon	LSV	1.20	
	Bismuth		1.22	
	Silver		1.06	

**Table 4:** Nickel content in red radish (*Raphanus sativus*).

Biosensor for Ni	SPEs	Technique	Nickel Content [mg/kg]	AAS [mg/kg]
Protein A-agarose	Carbon	CV	7.68	7.11
	Bismuth		8.21	
	Silver		7.42	
	Carbon	LSV	7.62	
	Bismuth		7.89	
	Silver		7.98	
Dimethylglyoxime-alginate	Carbon	CV	8.11	
	Bismuth		8.02	
	Silver		7.68	
	Carbon	LSV	7.56	
	Bismuth		7.28	
	Silver		7.72	
Dimethylglyoxime-benzophenone	Carbon	CV	7.82	
	Bismuth		7.51	
	Silver		8.68	
	Carbon	LSV	7.94	
	Bismuth		7.84	
	Silver		7.32	
Urease-alginate	Carbon	CV	7.62	
	Bismuth		7.46	
	Silver		7.18	
	Carbon	LSV	7.48	
	Bismuth		7.54	
	Silver		7.28	
Ethylenediamine-alginate	Carbon	CV	7.83	
	Bismuth		7.78	
	Silver		7.44	
	Carbon	LSV	8.20	
	Bismuth		8.12	
	Silver		8.36	

**Table 5:** Nickel content in white potato (*Solanum tuberosum*).

Biosensor for Ni	SPEs	Technique	Nickel Content [mg/kg]	AAS [mg/kg]
Protein A-agarose	Carbon	CV	2.98	2.35
	Bismuth		3.06	
	Silver		3.11	
	Carbon	LSV	2.85	
	Bismuth		2.93	
	Silver		2.96	
Dimethylglyoxime-alginate	Carbon	CV	2.17	
	Bismuth		3.08	
	Silver		3.29	
	Carbon	LSV	3.44	
	Bismuth		3.27	
	Silver		3.38	
Dimethylglyoxime-benzophenone	Carbon	CV	3.02	
	Bismuth		3.16	
	Silver		2.71	
	Carbon	LSV	2.92	
	Bismuth		3.10	
	Silver		3.01	
Urease-alginate	Carbon	CV	2.74	
	Bismuth		2.61	
	Silver		2.41	
	Carbon	LSV	2.49	
	Bismuth		2.72	
	Silver		2.81	
Ethylenediamine-alginate	Carbon	CV	2.79	
	Bismuth		2.98	
	Silver		2.92	
	Carbon	LSV	3.05	
	Bismuth		3.14	
	Silver		2.92	

## Data Availability

The data presented in this study are available upon request from the corresponding author.

## Supplementary Materials

The following are available online at https://www.mdpi.com/article/10.3390/bios11120519/s1, Figure S1: Voltammograms obtained for a nickel standard solution and Protein A-Agarose, Figure S2: Voltammograms obtained for a nickel standard solution and dimethylglyoxime immobilized with benzophenone, Figure S3: Voltammograms obtained for a nickel standard solution and ethylenediamine immobilized with alginate, Figure S4: Calibration curves obtained for a nickel standard solution and Protein A-Agarose, Figure S5: Calibration curves for a nickel standard solution and dimethylglyoxime immobilized with alginate, Figure S6: Calibration curves for a nickel standard solution and dimethylglyoxime immobilized with benzophenone, Figure S7: Calibration curves for a nickel standard solution and urease immobilized with alginate. Figure S8: Calibration curves obtained for a nickel standard solution and ethylenediamine immobilized with alginate.
