# Supplementary material for: Development, Optimization, Characterization, and Application of Electrochemical Biosensors for Detecting Nickel Ions in Food
Source: Biosensors (Basel). 2021 Dec 16;11(12):519. doi: 10.3390/bios11120519 (PMC8699131; doi:10.3390/bios11120519)
Supplement: Supplementary file 1 [file biosensors-11-00519-s001.zip › biosensors-1469179-supplementary.pdf]

biosensors

MDPI

*Article*

# Development, Optimization, Characterization and Application of Electrochemical Biosensors for Detecting Nickel Ions in Food

**Liliana Anchidin-Norocel [1], Wesley K. Savage [1,2,*], Gheorghe Gutt [3], Sonia Amariei [3]**

1 Faculty of Medicine and Biological Sciences, Stefan cel Mare University of Suceava, 720229 Suceava, Romania
2 Integrated Center for Research, Development and Innovation in Advanced Materials, Nanotechnologies, and Distributed Systems for Fabrication and Control, Stefan cel Mare University of Suceava, 720229 Suceava, Romania;
3 Faculty of Food Engineering, Stefan cel Mare University of Suceava, 720229 Suceava, Romania
* Correspondence wesley.savage@usm.ro

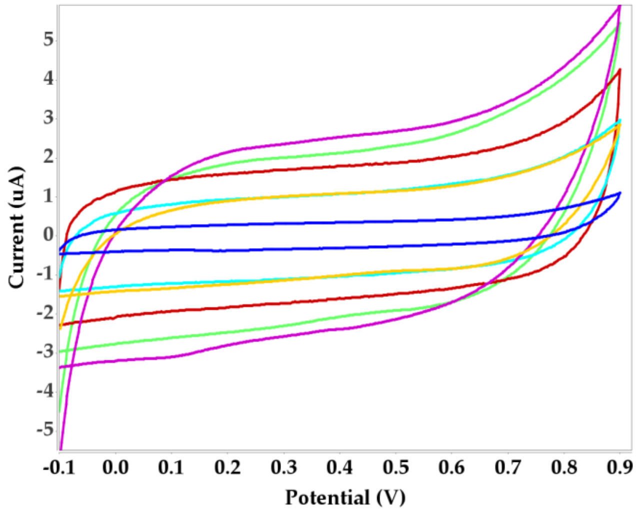


(**a**)

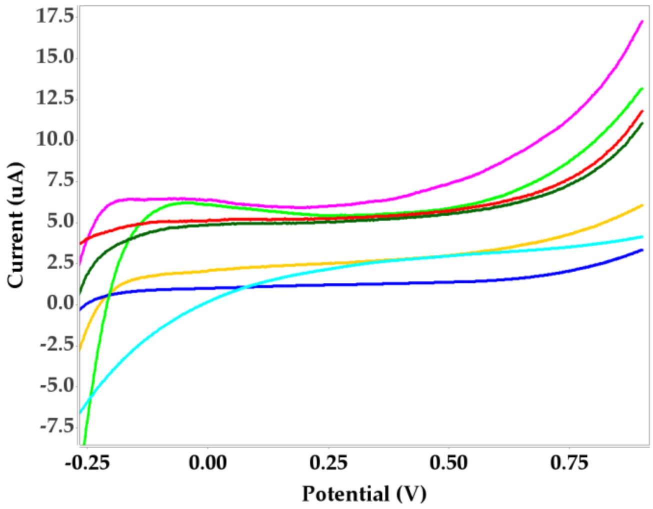


(**d**)

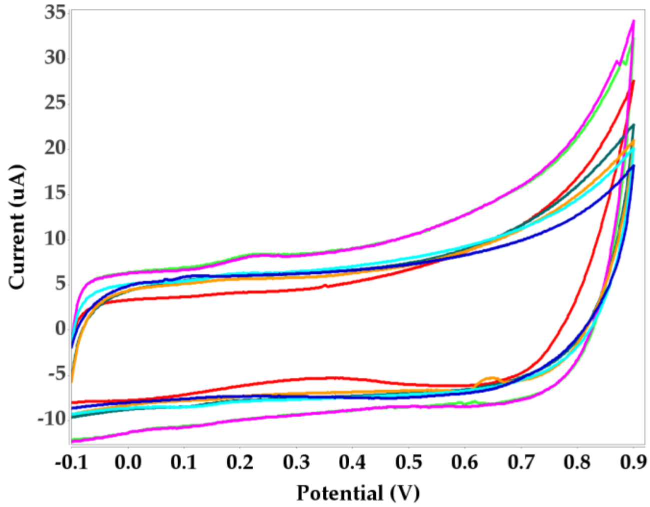


(**b**)

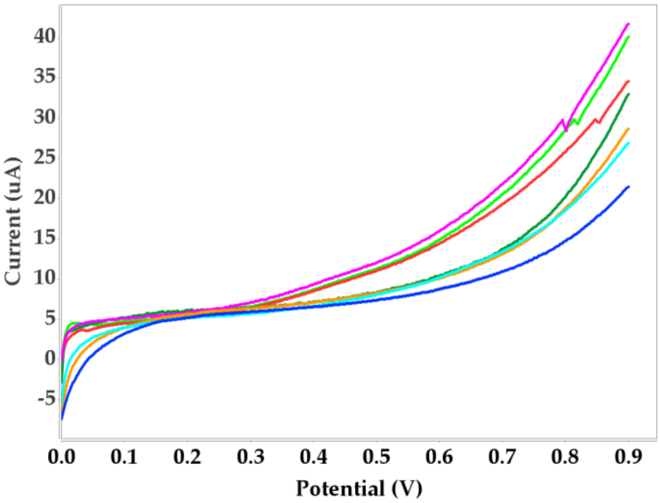


(**e**)

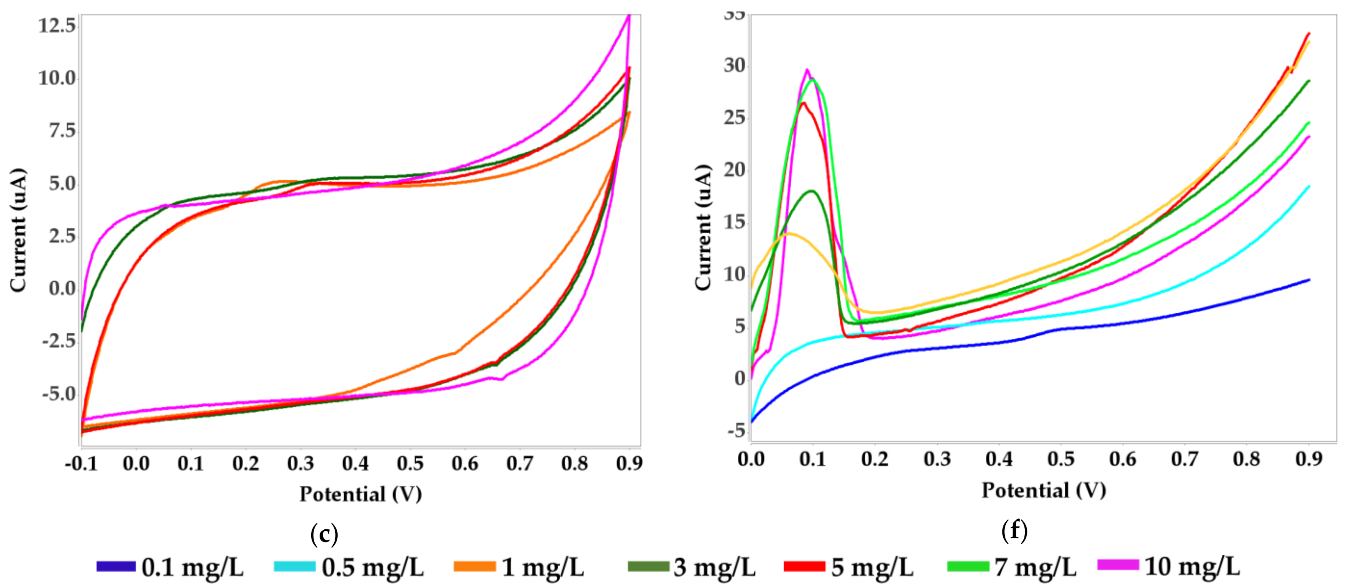


**Figure S1.** Voltammograms obtained for a nickel standard solution and Protein A-Agarose; Cyclic Voltammetry (CV) with Screen Printed Electrodes (SPEs) and (**a**) carbon, (**b**) bismuth, (**c**) silver; Linear Sweep Voltammetry (LSV) with SPEs and (**d**) carbon, (**e**) bismuth, (**f**) silver.

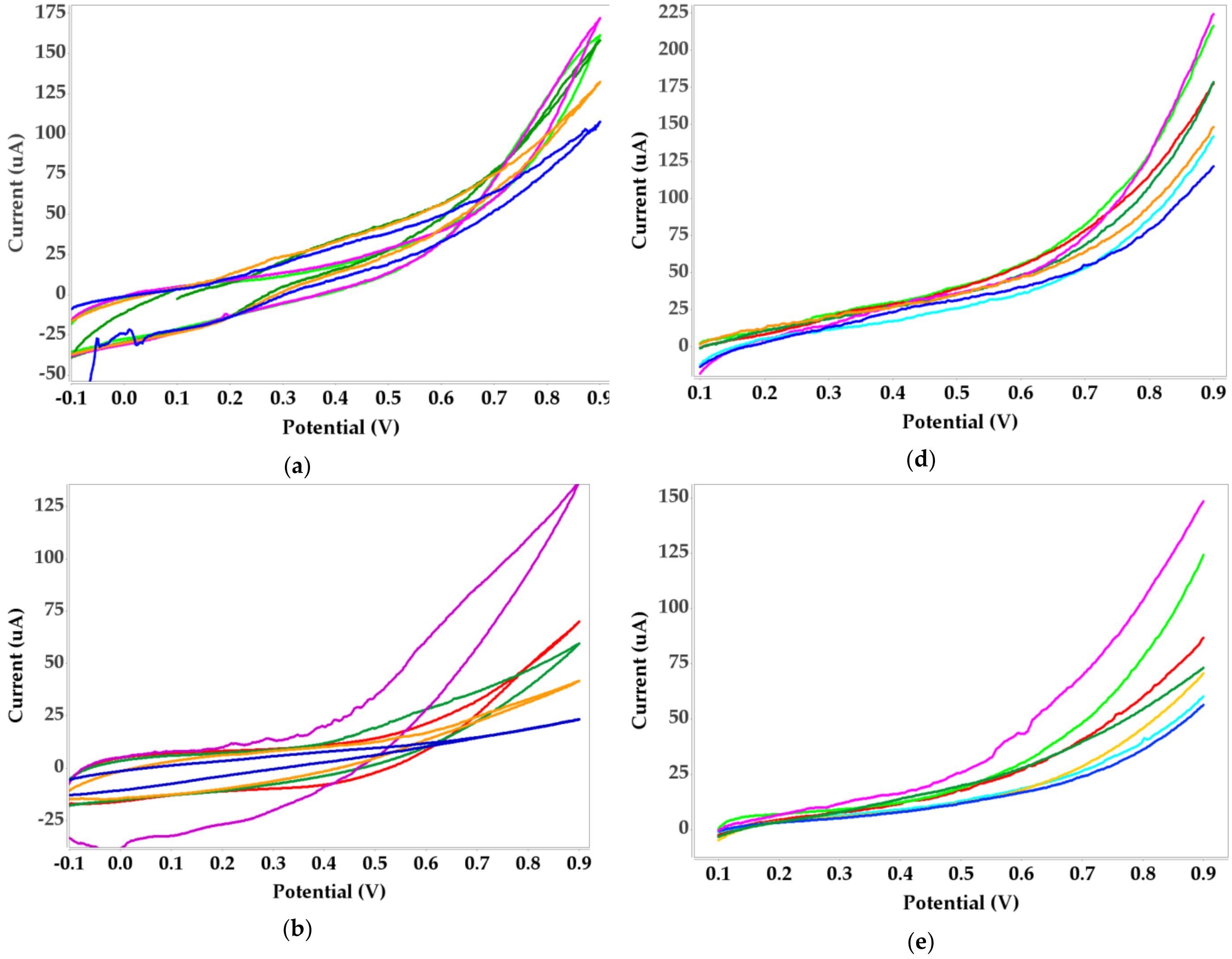

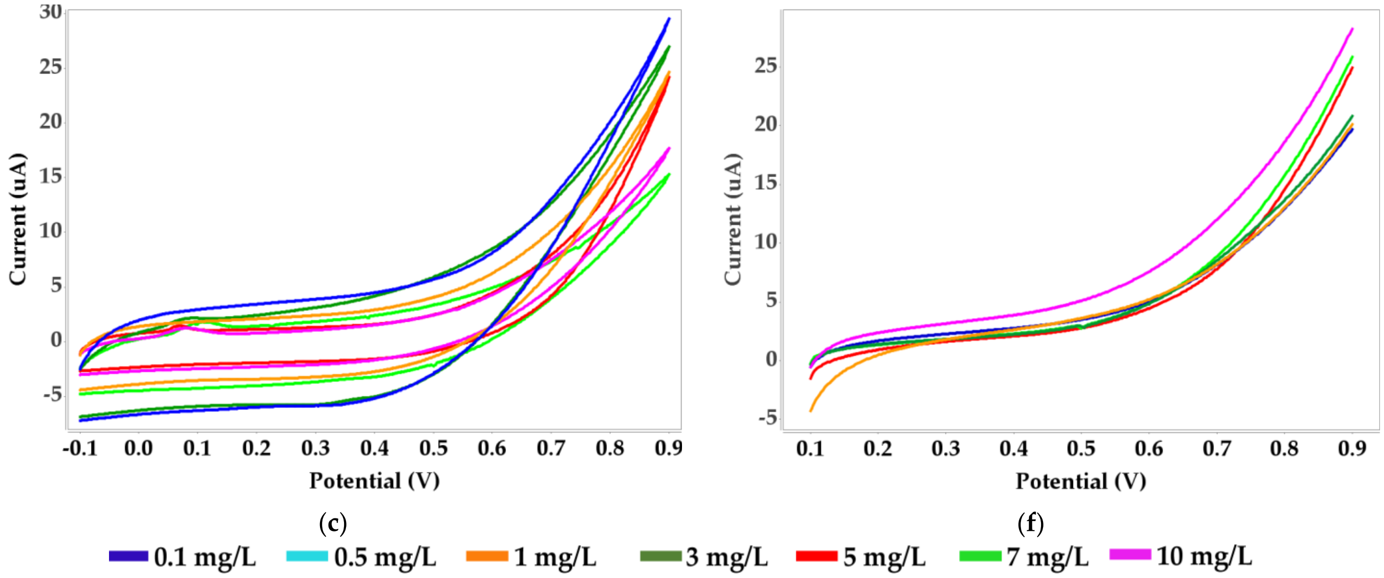


**Figure S2.** Voltammograms obtained for a nickel standard solution and dimethylglyoxime immobilized with benzophenone; CV with SPEs and (**a**) carbon, (**b**) bismuth, (**c**) silver; LSV with SPEs and (**d**) carbon, (**e**) bismuth, (**f**) silver.

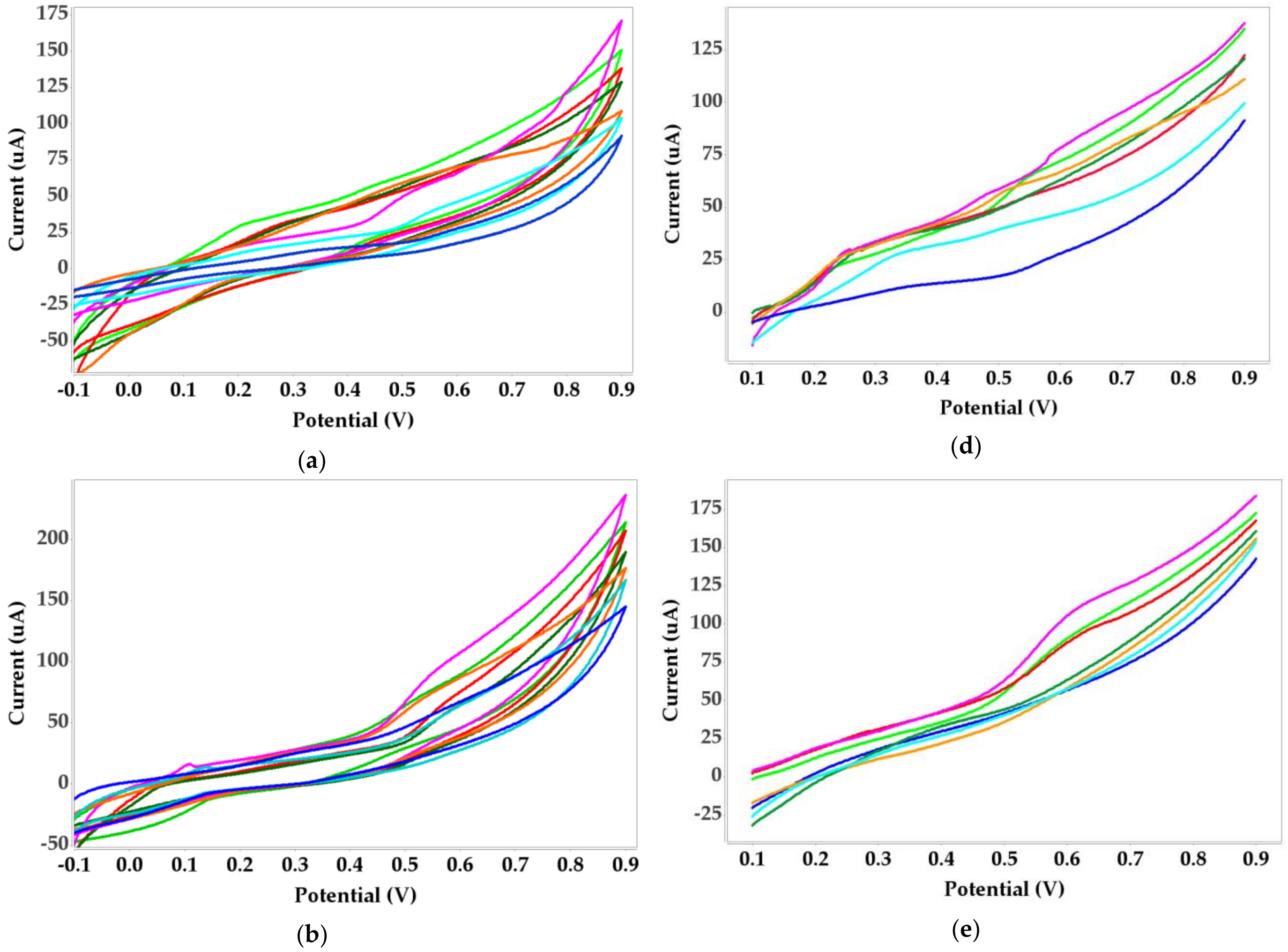

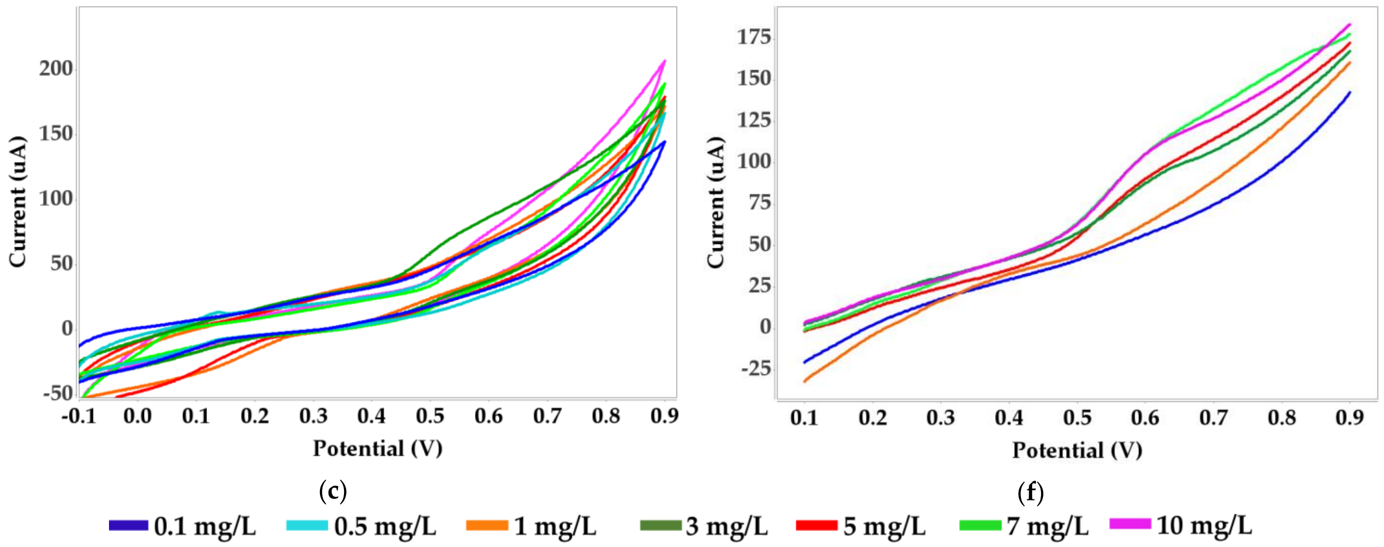


**Figure S3.** Voltammograms obtained for a nickel standard solution and ethylenediamine immobilized with alginate; CV with SPEs and (**a**) carbon, (**b**) bismuth, (**c**) silver; LSV with SPEs and (**d**) carbon, (**e**) bismuth, (**f**) silver.

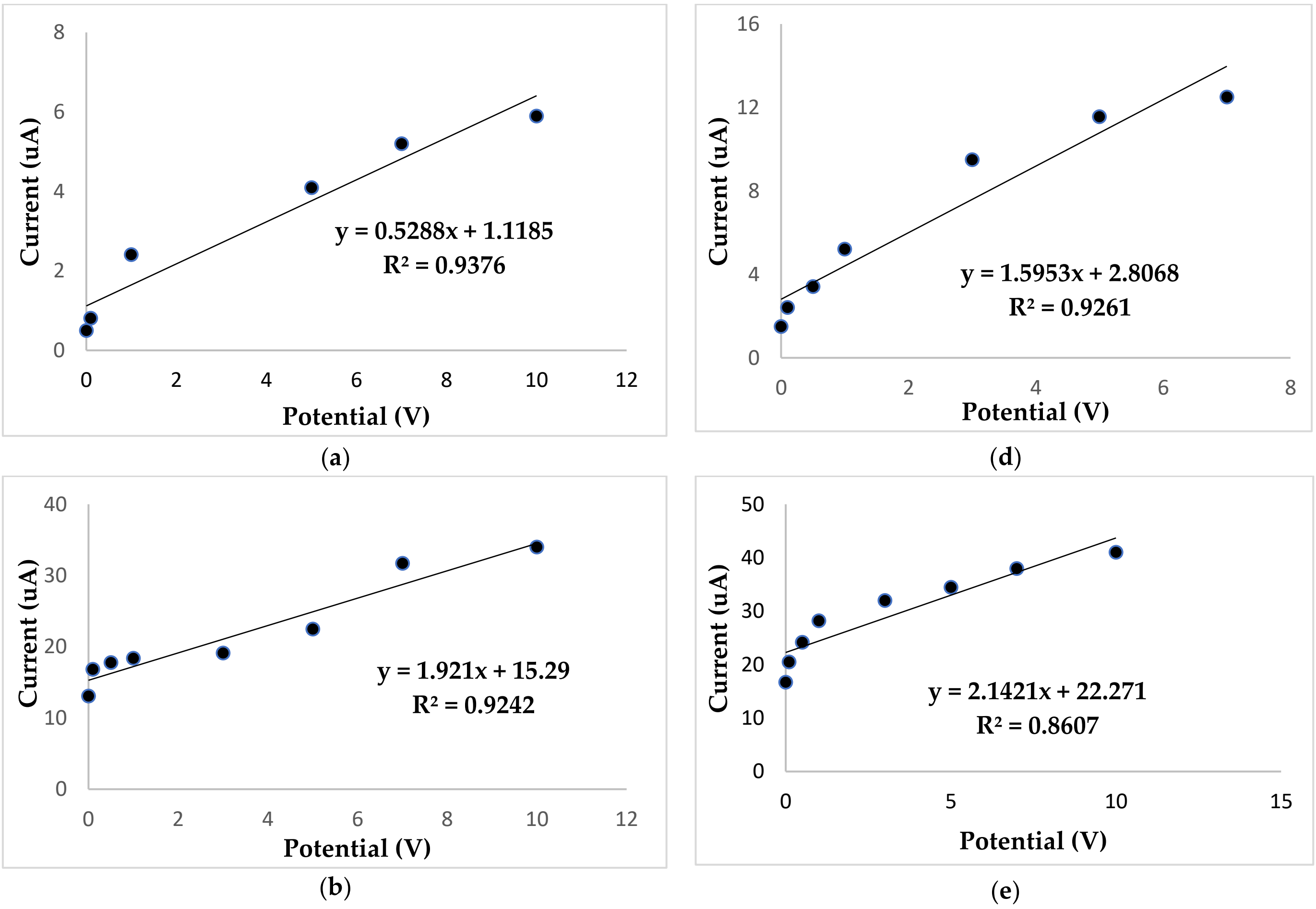

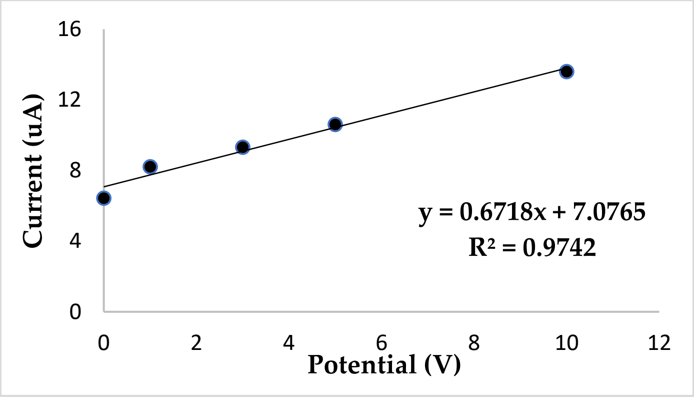


(**c**)

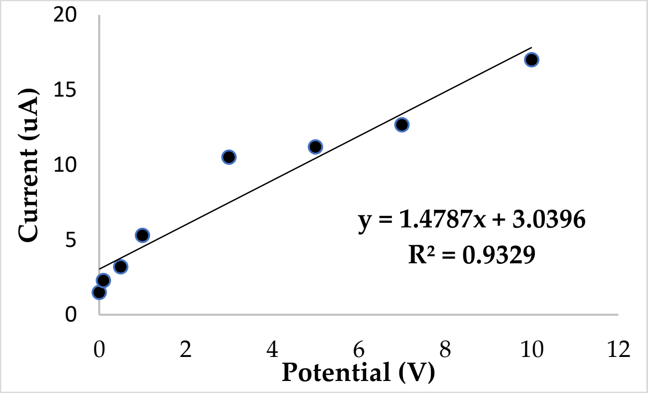


(**f**)

**Figure S4.** Calibration curves obtained for a nickel standard solution and Protein A-Agarose; CV with SPEs (**a**) carbon, (**b**) bismuth, (**c**) silver; LSV with SPEs (**d**) carbon, (**e**) bismuth, (**f**) silver.

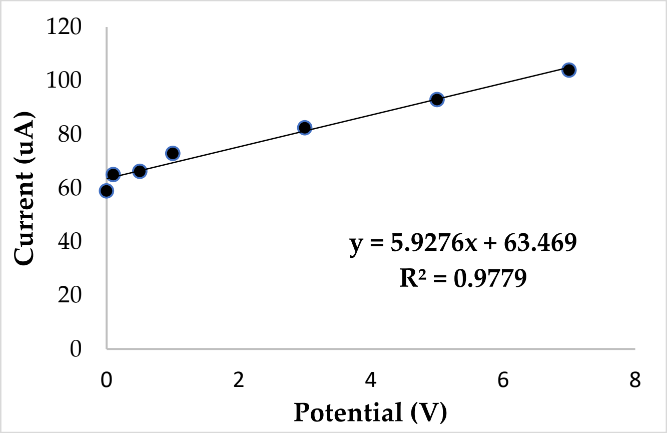


(**a**)

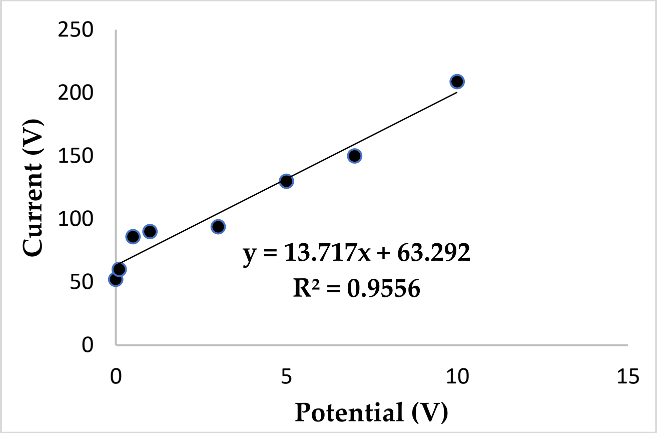


(**d**)

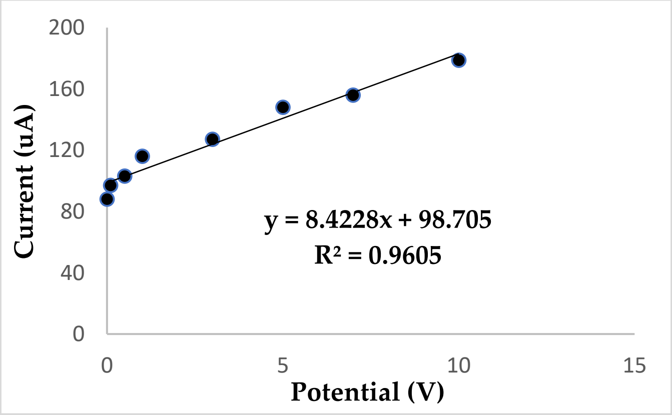


(**b**)

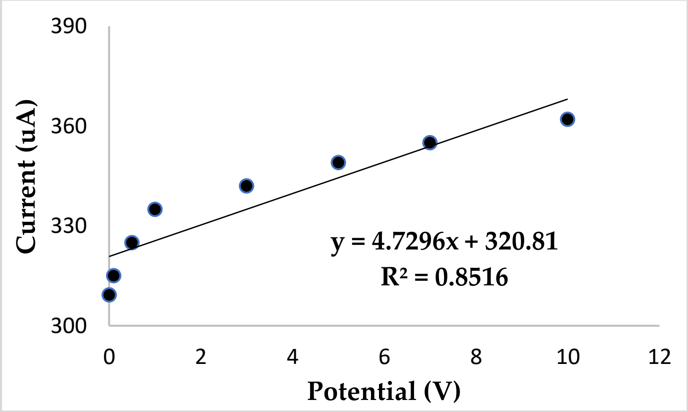


(**e**)

Figure in Main Text

(**c**)

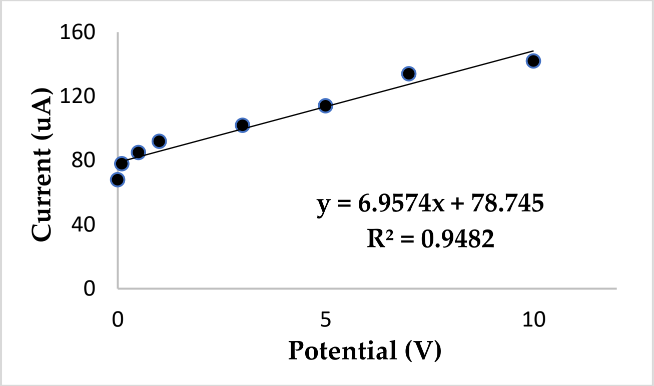


(**f**)

**Figure S5.** Calibration curves for a nickel standard solution and dimethylglyoxime immobilized with alginate (DMG-alginate); CV with SPEs (**a**) carbon, (**b**) bismuth, (**c**) silver (main text); LSV with SPEs (**d**) carbon, (**e**) bismuth, (**f**) silver.

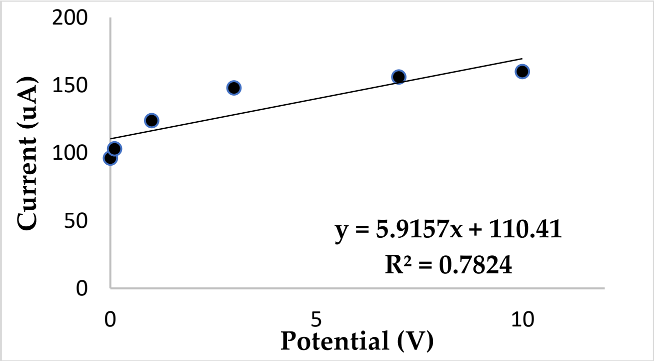


(**a**)

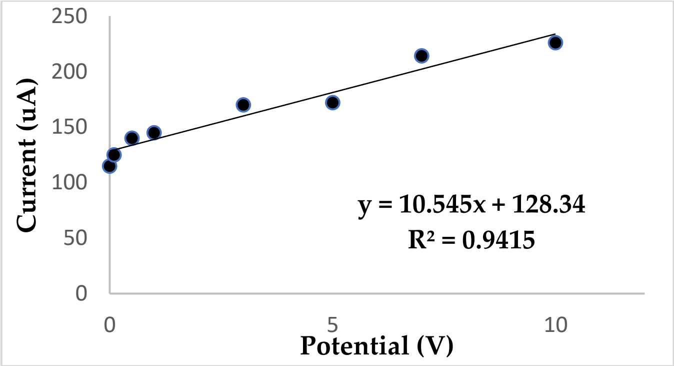


(**d**)

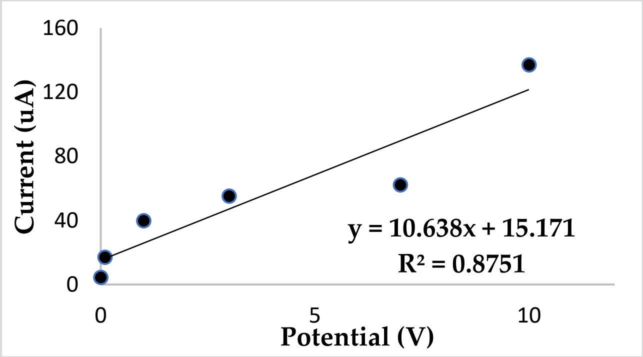


(**b**)

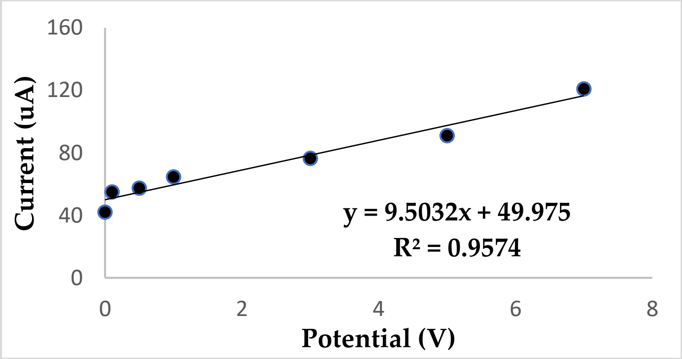


(**e**)

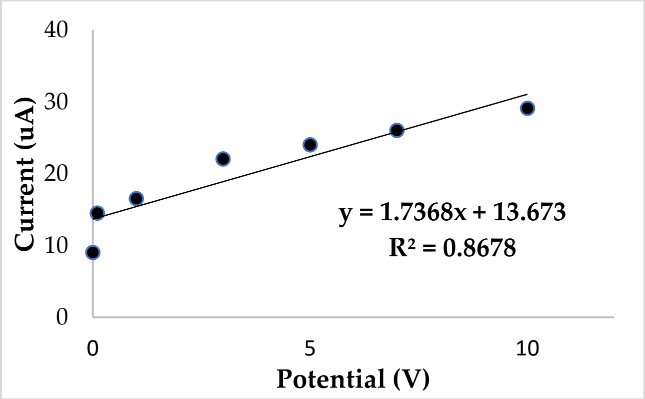


(**c**)

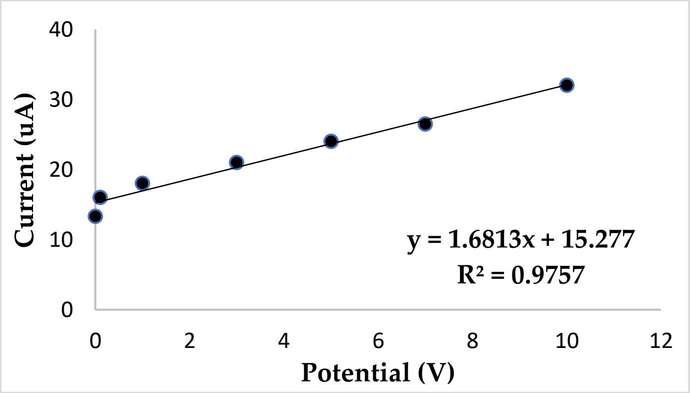


(**f**)

**Figure S6.** Calibration curves for a nickel standard solution and dimethylglyoxime immobilized with benzophenone; CV with SPEs (**a**) carbon, (**b**) bismuth, (**c**) silver; LSV with SPEs (**d**) carbon, (**e**) bismuth, (**f**) silver.

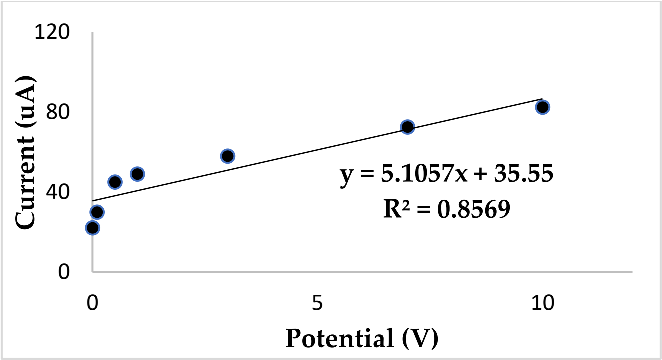


(**a**)

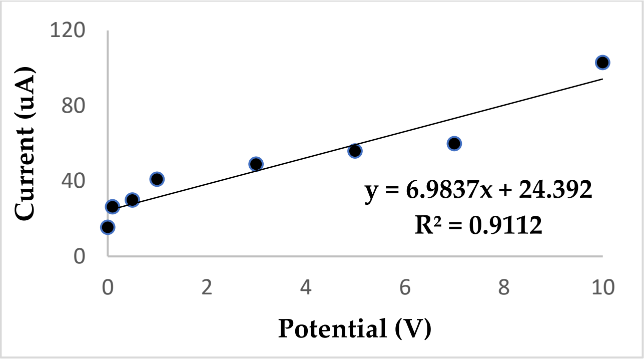


(**d**)

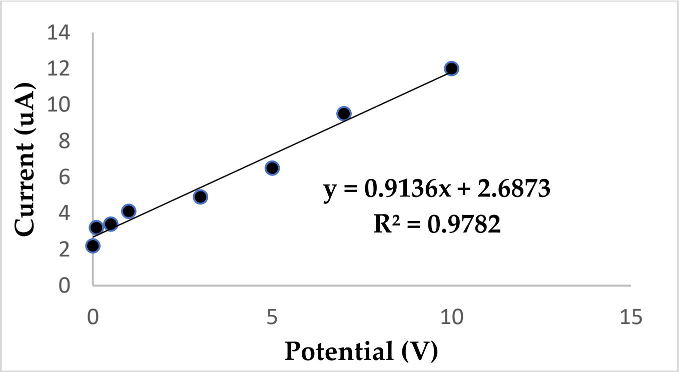


(**b**)

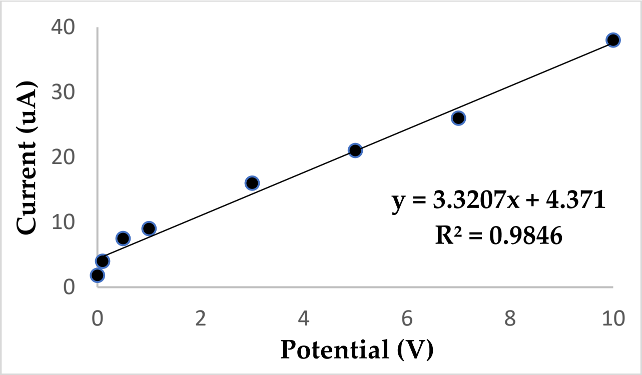


(**e**)

Figure in Main Text

(**c**)

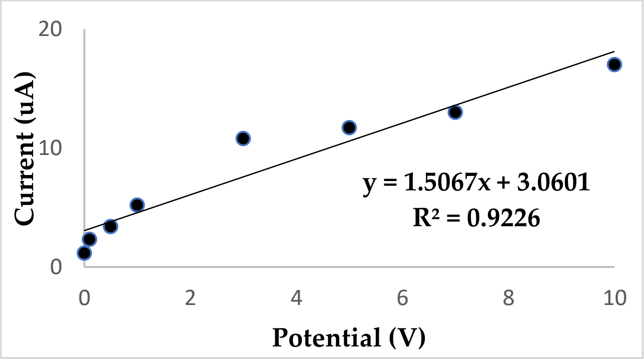


(**f**)

**Figure S7.** Calibration curves for a nickel standard solution and urease immobilized with alginate; CV with SPEs (**a**) carbon, (**b**) bismuth, (**c**) silver (main text); LSV with SPEs (**d**) carbon, (**e**) bismuth, (**f**) silver.

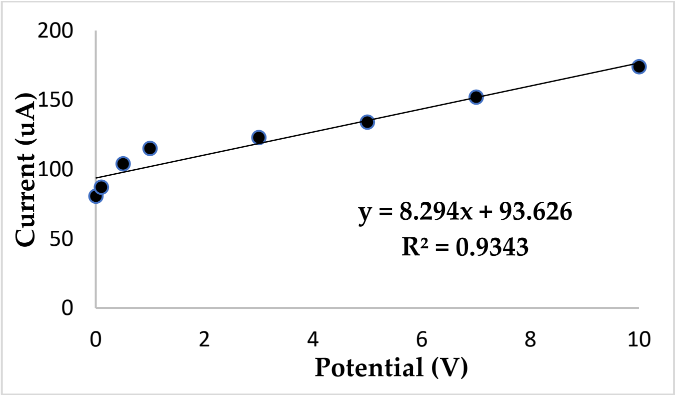


(**a**)

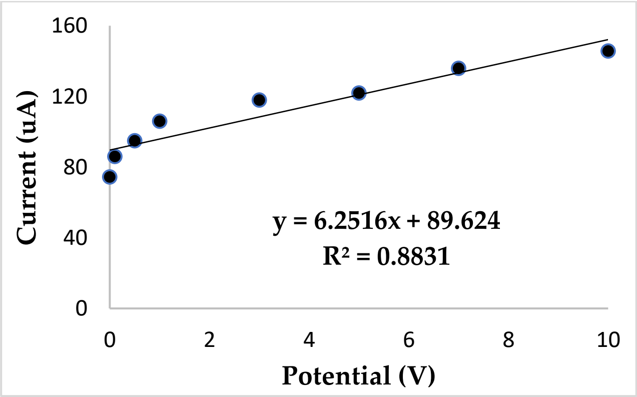


(**d**)

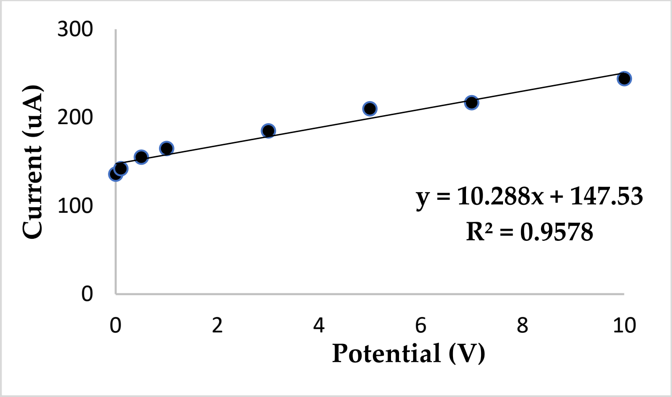


(**b**)

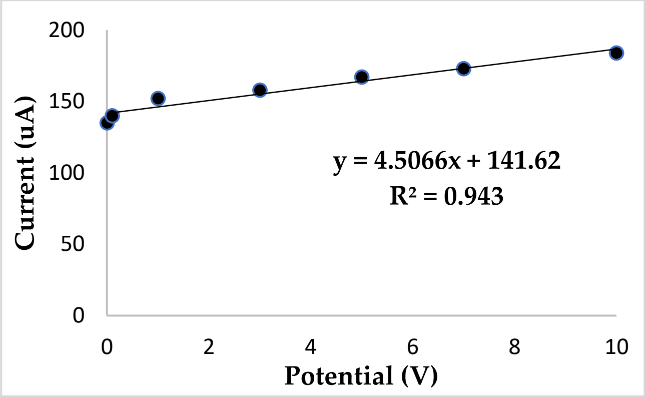


(**e**)

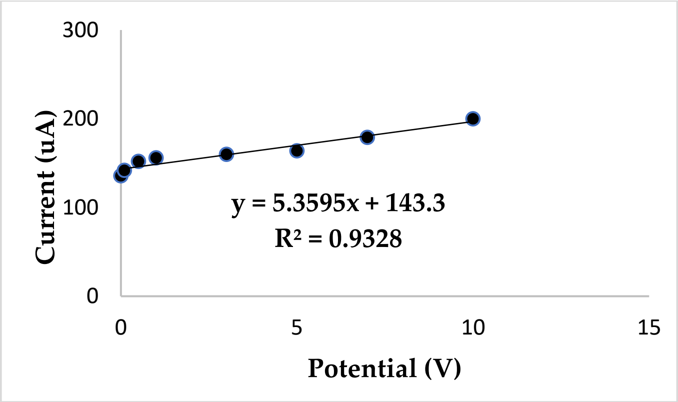


(**c**)

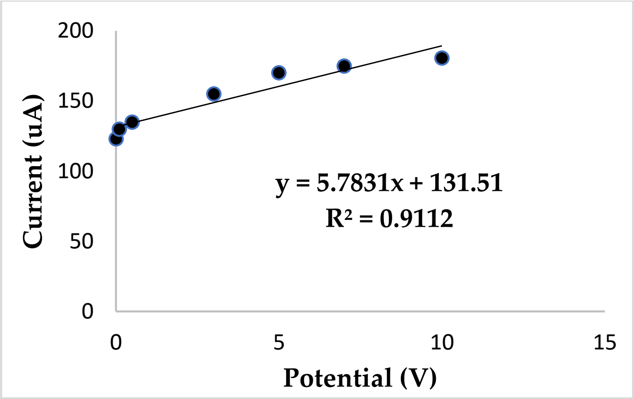


(**f**)

**Figure S8.** Calibration curves obtained for a nickel standard solution and ethylenediamine immobilized with alginate; CV with SPEs (**a**) carbon, (**b**) bismuth, (**c**) silver; LSV with SPEs (**d**) carbon, (**e**) bismuth, (**f**) silver.
